# HSPA5, a Host Cellular Heat-Shock Protein Required for Influenza a Virus Replication

**DOI:** 10.3390/ijms262210998

**Published:** 2025-11-13

**Authors:** Mahamud-ur Rashid, Tamanna Yasmin, Kevin M. Coombs

**Affiliations:** 1 Department of Medical Microbiology and Infectious Diseases, University of Manitoba, Room 543 Basic Medical Sciences Building, 745 Bannatyne Avenue, Winnipeg, MB R3E OJ9, Canada; rashidmm@myumanitoba.ca; 2 Manitoba Centre for Proteomics & Systems Biology, Room 799, 715 McDermot Avenue, Winnipeg, MB R3E 3P4, Canada; 3 Department of Biological Science, University of Manitoba, Winnipeg, MB R3T 2N2, Canada; yasmint@myumanitoba.ca; 4 Children’s Hospital Research Institute of Manitoba, Room 513, John Buhler Research Centre, 715 McDermot Avenue, Winnipeg, MB R3E 3P4, Canada

**Keywords:** HSPA5 1, influenza virus 2, EGCG 3, host dependency factor 3, replication

## Abstract

The Influenza A Virus (IAV) is known to hijack cellular proteins during its replication. IAV infection increases the expression of Heat-shock-protein family A (Hsp70) member 5 (HSPA5) in human cells, but its specific function in the viral life cycle remains unclear. This study aims to elucidate the function of HSPA5 in IAV replication, by implementing HSPA5 knockdown (KD) in A549 cells and assessing its impact on IAV’s viral protein translation, genomic RNA transcription, and the host cellular proteome. HSPA5 KD significantly reduced progeny virus release, although viral RNA levels were unaffected. Interestingly, levels of viral structural proteins increased in HSPA5 KD cells after infection. Treatment with HSPA5 inhibitor also suppressed IAV replication, confirming its role as a host dependency factor. Proteomic profiling revealed 116 proteins altered in wild-type cells and 223 in HSPA5 KD cells, with 32 uniquely dysregulated in wild-type and 139 unique to HSPA5 KD cells. In HSPA5 knockdown cells, the altered proteins were linked to pathways such as EIF2, EGF, PEDF, CNTF, IL-13, and G-protein receptor signaling, as well as to cellular processes like lymphocyte activation and regulation of immune and blood cell death, which were not affected in wild-type cells after IAV infection. Overall, this study suggests that HSPA5 contributes to late stages of IAV replication, likely assembly or maturation, and represents a promising target for antiviral drug development.

## 1. Introduction

Influenza A virus (IAV) is responsible for seasonal flu outbreaks, affecting nearly one billion individuals globally each year and leading to an estimated 3–5 million cases of severe illness and approximately 500,000 deaths [1]. Beyond seasonal outbreaks, IAV pandemics have historically caused catastrophic events, with an estimated 100 million deaths worldwide during the 20th century [2,3].

To date, 18 hemagglutinin (HA) and 11 neuraminidase (NA) subtypes of IAV have been identified based on antigenic diversity [4,5]. Among these, H1N1 and H3N2 are the predominant subtypes associated with seasonal influenza in humans [1]. The influenza virus genome is composed of eight negative-sense, single-stranded RNA segments [4]. Due to the error-prone nature of its replication, IAV undergoes frequent mutations, enabling it to develop resistance to antiviral agents and evade neutralizing antibodies, including those generated through vaccination [6]. Consequently, designing broadly effective vaccines remains a major challenge, and therapeutic options are often limited. Since viruses are obligate intracellular parasites, they depend on host cellular machinery for replication and for evading host immune defenses. Disruption of host proteins or signaling pathways that are essential for viral replication, and temporary disruption of which do not harm the cell, could therefore serve as an effective antiviral strategy. This highlights the importance of identifying host factors that are indispensable for IAV replication and clarifying their specific roles in the viral life cycle. Our earlier work using a 96-well siRNA screening assay demonstrated that silencing Heat Shock Protein Family A (Hsp70) member 5 (HSPA5) expression leads to reduced viral replication, suggesting that it may serve as a key host factor for influenza virus replication [7].

HSPA5, also referred to as BiP (Binding immunoglobulin protein) or glucose-regulated protein 78 (GRP78), is encoded by the *hspa5* gene located on human Chromosome 9 [8]. This gene is highly conserved across eukaryotic species and is ubiquitously expressed in nearly all human tissues [9]. The *hspa5* gene is expressed in all human nucleated cells, with nasal epithelial cells showing particularly high expression levels [10]. The protein consists of two main functional domains: the substrate-binding domain (SBD), which mediates interaction with polypeptides, and the nucleotide-binding domain (NBD), responsible for ATP binding and hydrolysis [11]. HSPA5 functions as a molecular chaperone in the endoplasmic reticulum (ER) lumen, where it assists in folding nascent peptides and ensuring their proper structural organization [12]. In addition, it facilitates polypeptide translocation into the ER lumen or membrane through an ATP-dependent process [13,14]. Importantly, HSPA5 plays a central role in regulating ER homeostasis by modulating the unfolded protein response (UPR) pathway during ER stress [15,16,17]. In addition, it has been implicated in regulating cell proliferation and apoptosis [18].

Recent studies have indicated that HSPA5 can directly bind the Zika virus E protein and may function as a host factor required for viral replication [19,20]. Similarly, its role is critical for the entry and replication of Japanese Encephalitis virus [21], dengue virus [22], and coxsackievirus A9 [23]. Notably, HSPA5 also contains a binding site for the SARS-CoV-2 spike protein [24], and its activity is essential for viral entry into host cells [25]. Inhibition of HSPA5 using epigallocatechin-3-gallate (EGCG), a green tea-derived polyphenol, has been shown to reduce replication of alpha-coronavirus (HCoV-229E) and beta-coronaviruses (HCoV-OC43 and SARS-CoV-2) in Rhabdomyosarcoma (RD) cells [25,26]. A recent study demonstrated that HSPA5 can directly interact with the hemagglutinin protein of IAV and may be involved in viral attachment and internalization [27], although the study was focused on avian strains of IAV on chicken fibroblast cells. Another study found that overexpression of HSPA5 may inhibit IAV replication by activating the IFN/JAK-STAT signaling pathways [28], indicating the role of HSPA5 in IAV replication is dynamic in nature, and may play more than one role.

Despite these findings, the precise role of HSPA5 in IAV genome replication, viral protein synthesis, or virion maturation during the replication cycle remains unclear. In addition, how HSPA5 participates in cellular signaling pathways and functions has not yet been systematically investigated. In the present study, we investigated how HSPA5 contributes to IAV replication by assessing the impact of HSPA5 knockdown (KD) on viral protein synthesis, genomic RNA transcription, and alterations in the host cellular proteome.

## 2. Results

### 2.1. Optimization of HSPA5 Knockdown by siRNA Treatment

**Figure 1:**
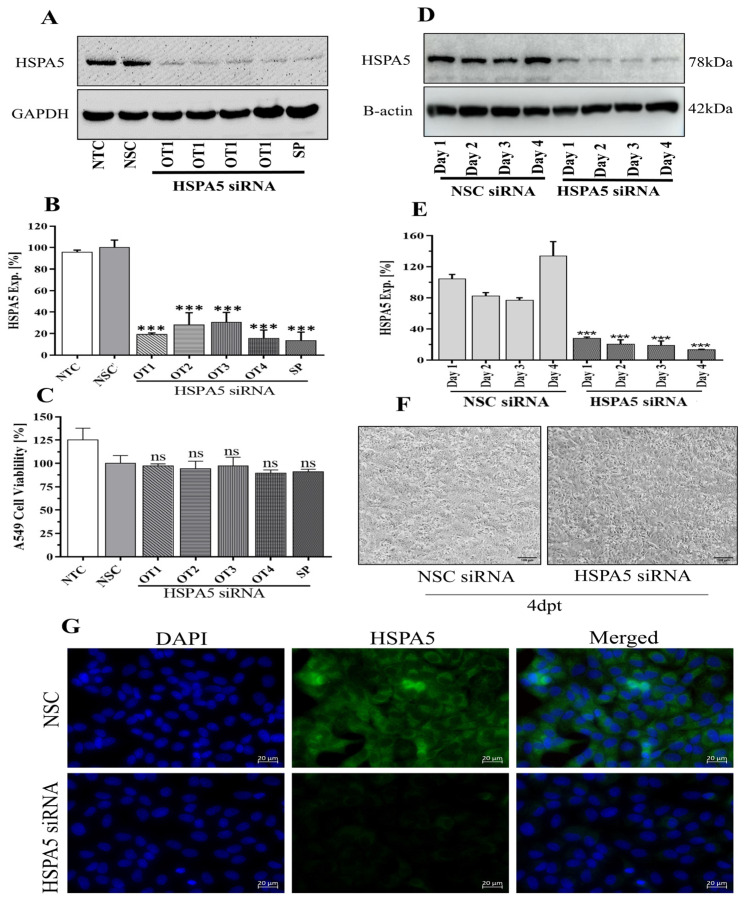
Optimization of HSPA5 silencing in A549 cells by siRNA transfection. (**A**) Western blot showing HSPA5 protein levels 48 h after transfection with 50 nM on-target (OT) or SMARTpool (SP) HSPA5 siRNA. (**B**) HSPA5 expression from densitometric quantification of Western blot images following OT and SP siRNA treatment. (**C**) Viability of A549 cells 48 h after siRNA transfection. (**D**) Time-course Western blot analysis of HSPA5 expression up to 4 days post-transfection (dpt) with 50 nM SP siRNA. (**E**) Densitometric quantification of HSPA5 expression over the 4-day period. (**F**) Representative bright-field microcopy images showing A549 cell morphology 4 dpt following 50 nM HSPA5 siRNA transfection (scale bar = 100 µm). (**G**) Immunofluorescence image confirmation of HSPA5 knockdown (scale bar = 20 µm). Cell nuclei were stained with DAPI. Exp = expression, NSC = non-silencing control, NTC = non-treated control. *** *p* < 0.001.

To knockdown HSPA5 expression, A549 cells were transfected with 50 nM of either four individual on-target (OT) siRNAs or a pooled set (SmartPool, SP) targeted against HSPA5 for 48 h. Both OT and SP siRNA treatments led to a significant decrease in HSPA5 protein levels (Figure 1A,B) without affecting overall cell viability (Figure 1C). To assess the stability of knockdown, A549 cells were further treated with 50 nM HSPA5 SP siRNA for up to four days. Protein expression was gradually reduced to 28%, 20%, 18%, and 13% on days 1, 2, 3, and 4, respectively (Figure 1D,E). After four days of transfection, cell morphology was not significantly different between non-silencing control (NSC) and HSPA5 siRNA treated groups under light microscope (Figure 1F). Knockdown of HSPA5 was further verified by immunofluorescence microscopy (Figure 1G).

### 2.2. Impact of HSPA5 KD on IAV Replication

**Figure 2:**
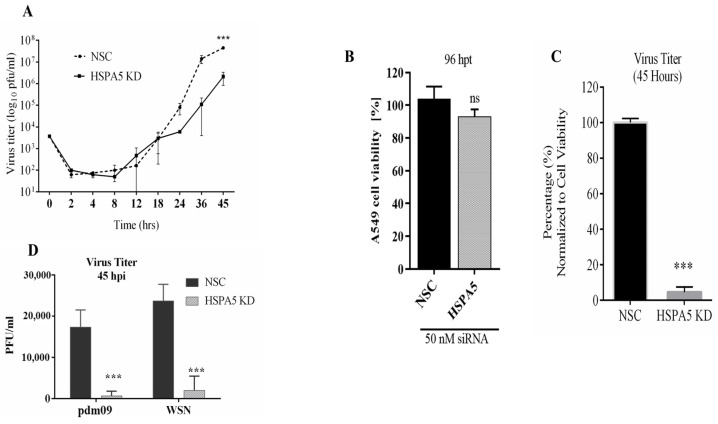
HSPA5 is an essential protein during IAV replication. A549 cells were transfected with either non-silencing control (NSC) siRNA or HSPA5-targeting siRNA (HSPA5 KD) for 48 h, followed by infection with the IAV PR8 strain at a multiplicity of infection (MOI) of 0.01. Virus titers was monitored by collecting cell supernatants at various intervals from 0 to 45 h post-infection (hpi). Similar experiments were done using the pdm09 and WSN IAV strains, with supernatants collected at 45 hpi. Virus quantification was performed via plaque assay. (**A**) Kinetics of IAV (PR8 strain) titer in the supernatant of HSPA5 KD cells compared to NSC cells. (**B**) Cell viability, measured by WST-1 assay 96 h after siRNA transfection. (**C**) Percentage of virus titer in HSPA5 KD cell supernatant at 45 hpi, normalized to NSC and adjusted for cell viability. (**D**) Effect of HSPA5 knockdown on replication of pdm09 and WSN IAV strains. Statistical significance: *** *p* < 0.001.

To assess the role of HSPA5 KD in IAV replication, A549 cells were transfected with either an NSC-siRNA or HSPA5-targeting siRNA for 48 h prior to infection with the PR8 strain. Supernatants were collected at multiple time points up to 45 hpi, and progeny virus production was quantified by plaque assay. Knockdown of HSPA5 led to a significant decrease in viral yield at 45 hpi (Figure 2A). Cell viability was not significantly affected by HSPA5 depletion (Figure 2B). When viral titers were normalized to viable cell counts, HSPA5 KD resulted in approximately a 95% reduction in progeny virus compared with control cells (Figure 2C). This inhibitory effect was not limited to the PR8 strain, as similar reductions in replication were also observed for pdm09 and WSN strains (Figure 2D).

### 2.3. Inhibition of HSPA5 by Epigallocatechin-3-Gallate (EGCG) Suppresses IAV Replication

**Figure 3:**
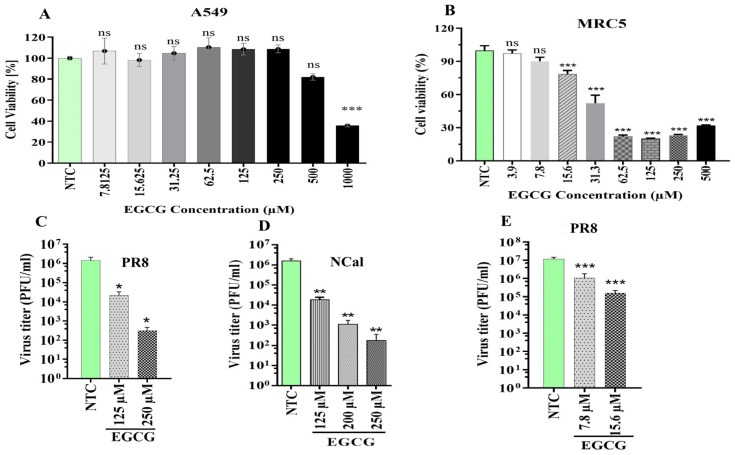
Inhibition of HSPA5 by EGCG suppresses IAV replication. EGCG cytotoxicity was assessed in (**A**) A549 and (**B**) MRC5 cells using the WST-1 assay across different concentrations. To evaluate its effect on viral replication, A549 and MRC5 cells were pre-treated with EGCG and infected with PR8 or NCal (MOI 0.01), with the drug also present in the overlay medium. Viral samples were collected at 45 hpi. Panels show impact of EGCG on (**C**) PR8 and (**D**) NCal in A549 cells, and (**E**) PR8 in MRC5 cells. Green bars indicate NTC controls; grayscale bars show increasing EGCG concentrations. ns = not significant. * *p* < 0.05, ** *p* < 0.01, *** *p* < 0.001.

Silencing of HSPA5 expression resulted in a significant decrease in IAV replication, highlighting the critical role of this chaperone as a host factor for IAV replication. To further examine whether inhibition of HSPA5 by EGCG, an established HSPA5 inhibitor, could impact IAV propagation, cytotoxicity assays were first conducted in A549 (Figure 3A) and MRC5 (Figure 3B) cell lines and concentrations that caused less than 20% cytotoxicity were selected for antiviral testing: 125, 200, and 250 μM for A549 cells, and 7.8 and 15.6 μM for MRC5 cells. EGCG treatment in A549 cells significantly reduced replication of the PR8 (Figure 3C) and NCal (Figure 3D) IAV strains. Interestingly, even lower EGCG concentrations significantly inhibited PR8 replication in MRC5 cells (Figure 3E).

### 2.4. Effect of HSPA5 Silencing on IAV Viral Protein Synthesis and vRNA Transcription

**Figure 4:**
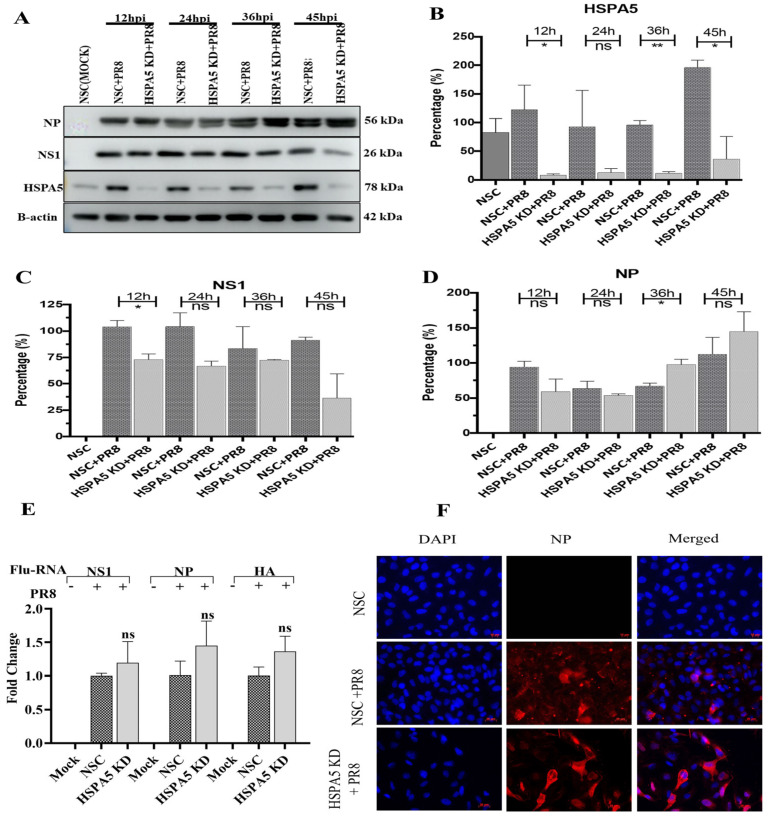
Effect of HSPA5 silencing on IAV viral protein synthesis and vRNA transcription. A549 cells were transfected with HSPA5 siRNA (HSPA5 KD) or non-silencing control (NSC) for 48 h and infected with IAV-PR8 at an MOI of 3. Protein samples collected at 12, 24, 36, and 48 hpi were analyzed by Western blot. At 24 hpi, cells were fixed for NP immunofluorescence imaging, and viral RNAs were isolated for qRT-PCR. (**A**) Western blot of NP and NS1 proteins in HSPA5 KD cells. (**B**–**D**) Densitometric analysis of HSPA5 (**B**), NS1 (**C**), and NP (**D**) expression. (**E**) Relative NS1, NP, and HA vRNA levels in HSPA5 KD versus NSC cells. (**F**) NP immunofluorescence showing enhanced signal in HSPA5 KD cells. ns = not significant. * *p* < 0.05, ** *p* < 0.01.

To investigate the effect of HSPA5 silencing on viral protein synthesis, HSPA5 KD and NSC A549 cells were infected with PR8 at an MOI of 3. Infected cells were collected at 12, 24, 36, and 48 hpi and IAV NS1 and NP protein levels were analysed by Western blot (Figure 4A). Efficient knockdown of HSPA5 was confirmed by Western blot (Figure 4B). NS1 expression was significantly reduced at 12 hpi in HSPA5 KD cells, while NP levels were significantly elevated at 36 hpi compared to controls (Figure 4C,D). To evaluate the impact of HSPA5 KD on virus RNA (vRNA) transcription, HSPA5 KD and NSC cells were infected at MOI 3 and harvested at 24 hpi for vRNA extraction. Following reverse transcription, qPCR was performed targeting NS1, NP, and HA vRNAs. HSPA5 knockdown did not significantly alter the transcription of any of the vRNAs analyzed (Figure 4E). To visualize viral protein accumulation, HSPA5 KD and control cells were infected with PR8 at MOI 3 and analyzed by NP-specific immunofluorescence at 24 hpi. A significant increase in NP signal intensity was observed in HSPA5 KD cells compared with controls (Figure 4F).

### 2.5. Impact of HSPA5 Knockdown on Proteome Regulation During IAV Infection

To investigate the role of HSPA5 in IAV replication, we examined how PR8 infection altered the proteomic profile of HSPA5 KD cells. Proteomic changes were quantified using SomaScan, a high-throughput platform capable of simultaneously measuring 1307 proteins across up to 92 samples [29]. Comparisons were made between PR8-infected wild-type A549 cells and their non-silencing control (NSC + PR8 vs. NSC), as well as between PR8-infected HSPA5 KD cells and uninfected HSPA5 KD cells (HSPA5 KD + PR8 vs. HSPA5 KD). Analysis revealed significant dysregulation of 218 proteins in response to PR8 infection and 503 proteins following HSPA5 KD with PR8 infection (Table 1).

Applying thresholds of ≥1.3- or ≤−1.3-fold change along with a *p*-value < 0.05, we identified 116 proteins (31 upregulated and 85 downregulated) in response to PR8 infection, and 223 proteins (78 upregulated and 145 downregulated) in HSPA5 KD + PR8–infected cells for subsequent bioinformatic analysis. Proteins showing ≥ 1.5-fold changes in either direction are summarized in Table 2.

**Figure 5:**
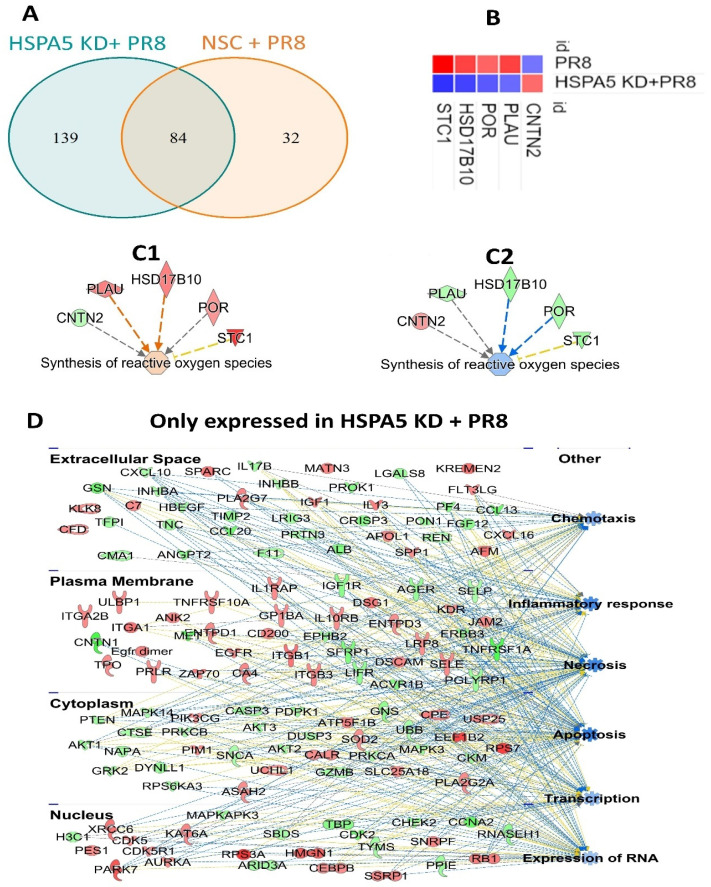
Effect of HSPA5 knockdown on the proteomic profile of A549 cells during IAV infection. (**A**) Venn diagram illustrating proteins altered in PR8-infected NSC and HSPA5 KD A549 cells. (**B**) Heatmap of differentially expressed proteins, where red indicates upregulation and blue indicates downregulation. (**C1**,**C2**) Predicted association of these proteins with reactive oxygen species (ROS) generation in wild-type and HSPA5 KD + PR8 cells. (**D**) Functional protein–protein interaction network of molecules uniquely affected in HSPA5 KD + PR8 cells. Upregulated proteins are shown in red, downregulated in green; orange and blue indicate predicted activation and inhibition, respectively.

While expression of 84 proteins was dysregulated in both wild-type and HSPA5 KD cells, 139 and 32 proteins were uniquely altered in HSPA5 KD and wild-type cells, respectively, following PR8 infection (Figure 5A). Notably, five proteins showed opposite patterns of regulation in HSPA5 KD cells compared to wild-type cells after PR8 infection. These included contactin 2 (CNTN2), plasminogen activator (PLAU), cytochrome P450 oxidoreductase (POR), hydroxysteroid 17-beta dehydrogenase 10 (HSD17B10), and stanniocalcin 1 (STC1) (Figure 5B). Expression of these proteins was associated with activation of reactive oxygen species synthesis in wild-type cells but inhibition in HSPA5 KD cells during viral infection (Figure 5C1,C2). The 139 proteins that were specifically dysregulated in HSPA5 KD + PR8 infected cells were further analyzed using Ingenuity Pathway Analysis (IPA). This analysis linked the altered proteins to inhibition of chemotaxis, inflammatory responses, necrosis, apoptosis, transcription, and RNA expression (Figure 5D).

**Figure 6:**
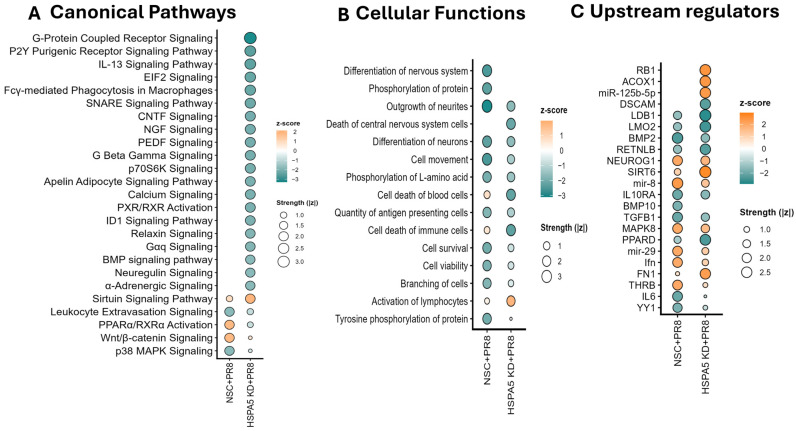
Impact of HSPA5 KD on cellular functions, signaling pathways, and upstream regulators in A549 cells during IAV infection. (**A**) Signaling pathways significantly dysregulated only in NSC + PR8 or HSPA5 KD + PR8 cells. (**B**) Cellular functions significantly dysregulated only in NSC + PR8 or HSPA5 KD + PR8 cells after IAV-PR8 infection. (Cancer related functions were excluded in the graph). (**C**) Topmost upstream regulators predicted to be significantly dysregulated only in NSC + PR8 or HSPA5 KD + PR8 cells. Full list of signaling pathways, cellular functions, and upstream regulators that were differentially regulated between wild-type and HSPA5 KD cells after IAV infection are provided in Table S2.

Bioinformatic analysis of differentially expressed proteins in NSC + PR8 and HSPA5 KD + PR8 cells using IPA indicated that several signaling pathways, biological functions, and upstream regulators were differentially regulated in HSPA5 KD cells relative to wild-type controls after influenza infection. In wild-type cells, activation of the PPARα/RXRα and Wnt/β-catenin signaling pathways was predicted, whereas leukocyte extravasation and p38 MAPK signaling were significantly suppressed. These regulatory changes were absent from HSPA5 KD cells. Conversely, signaling pathways such as G-protein coupled receptor, P2Y purinergic receptor, IL-13, EIF2, and those associated with SNARE, PEDF, CNTF, and NGF were significantly affected in HSPA5 KD cells but remained unchanged in PR8-infected wild-type cells (Figure 6A).

Additional IPA analysis showed that several cellular functions impacted in wild-type cells, such as protein phosphorylation, cell survival, cell motility, and cell branching, were not significantly affected in HSPA5 KD cells after PR8 infection. Interestingly, neuron-related processes, including neuronal differentiation and growth, were strongly inhibited in wild-type cells but remained unaffected in HSPA5 KD cells. Moreover, activation of lymphocytes and inhibition of immune cell death were predicted from the dysregulated proteins in HSPA5 KD cells, whereas these processes showed no significant changes in wild-type cells after PR8 infection (Figure 6B).

IPA identified 53 upstream regulators (30 activated and 23 inhibited) that were significantly dysregulated in HSPA5 KD cells but remained unchanged in wild-type cells, while 23 upstream regulators (15 activated and 8 inhibited) were altered in wild-type cells but not in HSPA5 KD cells (Table S2). In wild-type cells, the most inhibited upstream regulators included bone morphogenetic protein 2 (BMP2), transforming growth factor beta 2 (TGFB2), interleukin-6 (IL6), interleukin-10 receptor alpha (IL10RA), Yin Yang 1 (YY1), and bone morphogenetic protein 10 (BMP10), whereas the most activated regulators were interferon (IFN), microRNA-29 (miR-29), mitogen-activated protein kinase 8 (MAPK8), thyroid hormone receptor beta (THRB), neurogenin 1 (NEUROG1), and microRNA-8 (miR-8). In contrast, the top inhibited regulators in HSPA5 KD cells were LIM domain-binding protein 1 (LDB1), LIM domain only protein 2 (LMO2), peroxisome proliferator-activated receptor delta (PPARD), resistin-like beta (RETNLB), and Down syndrome cell adhesion molecule (DSCAM), while the most activated were fibronectin 1 (FN1), microRNA-125b-5p (miR-125b-5p), acyl-CoA oxidase 1 (ACOX1), retinoblastoma 1 (RB1), and sirtuin 6 (SIRT6) (Figure 6C). Full list of signaling pathways, cellular functions, and upstream regulators that were differentially regulated between wild-type and HSPA5 KD cells after IAV infection are provided in Table S2.

## 3. Discussion

### 3.1. HSPA5 KD Alters IAV-Mediated Host Proteomic Responses

Using proteomic analysis, we identified five proteins (CNTN2, PLAU, HSD17B10, POR, and STC1) (Figure 5B) that were significantly differentially regulated in wild-type and HSPA5 KD cells following IAV infection. These proteins were associated with reactive oxygen species (ROS) synthesis. In our previous work, we showed that IAV can suppress the NRF2-mediated oxidative stress response [7]. Other studies have also reported that IAV H5N1 serotype infection induces ROS production [30]. Among these proteins, PLAU expression was also found to be increased during HPV infection [31,32] and SARS-CoV-2 infection [33]. STC1 has been proposed as a serum biomarker and potential therapeutic target for hepatitis B virus (HBV) associated liver damage [34] and has been linked with fatigue severity in SARS-CoV-2 infection [35]. By contrast, POR, CNTN2, and HSD17B10 have not previously been associated with viral diseases. However, the role of HSPA5 in regulating ROS during IAV infection has not been described before. Further studies are required to better understand the role of HSPA5 in ROS-mediated regulation of IAV replication.

Analysis of the 139 proteins uniquely altered in HSPA5 KD cells predicted inhibition of chemotaxis, inflammatory responses, necrosis, apoptosis, transcription, and RNA expression. Influenza infection typically activates chemotaxis and inflammatory responses [36,37,38]. Apoptosis and necrosis are also well-established mechanisms of influenza-induced cell death [39,40,41,42,43]. Although we did not observe a significant impact of HSPA5 KD on viral RNA replication (Figure 4E), proteins associated with transcription and RNA expression were altered in HSPA5 KD cells during IAV infection. Overall, the proteomic analysis suggests that HSPA5 may play a role in regulating chemotaxis, inflammatory responses, and cell death by necrosis and apoptosis during influenza infection, demanding further investigation.

### 3.2. HSPA5 KD Causes Differential Regulation of Cellular Functions and Signaling Pathways During IAV Infection

Functional analysis of the proteins using IPA predicted that phosphorylation of tyrosine and L-amino acids was significantly reduced in wild-type A549 cells during IAV infection but remained unaffected in HSPA5 KD cells (Figure 6B). Previous studies have reported tyrosine dephosphorylation during H1N1 swine flu infection [44], which aligns with our findings. However, other studies have shown that tyrosine phosphorylation facilitates nuclear transport and M1 protein assembly during IAV replication [45,46,47]. In this study, we also observed that the IAV structural protein NP accumulated in HSPA5 KD cells, which may indicate an effect on NP assembly mediated by tyrosine phosphorylation. The precise role of HSPA5 in relation to tyrosine and L-amino acid phosphorylation requires further investigation.

In HSPA5 KD cells, several signaling pathways were significantly inhibited but not affected in wild-type cells, including G-protein coupled receptor, EIF2, calcium, NGF, PEDF, and IL-13 signaling (Figure 6A). Influenza virus uses G-protein coupled receptor proteins for their replication [48]. EIF2 signaling is exploited by different viruses, although its role in influenza virus replication is not clearly understood [49]. Calcium signaling has been identified as a potential antiviral therapeutic target for HBV infection [50,51] and is activated during herpes simplex virus infection [52]. This pathway is used by a wide range of viruses for entry, replication, and release [53]. Calcium signaling is also important for IAV internalization in infected cells [51]. NGF signaling has been shown to be altered by herpes simplex virus-2 [54] and respiratory syncytial virus [55]. PEDF signaling is not well characterized in viral infections but was found to be inhibited in HSPA5 KD cells after IAV infection. IL-13, an inflammatory cytokine, enhances immune cell recruitment at the site of infection [56] during IAV infection and has been linked to increased susceptibility to secondary infections [57]. However, the role of HSPA5 in regulating these pathways during influenza virus replication remains unknown.

The top upstream regulators uniquely predicted to be affected by HSPA5 KD after IAV infection included LDB1, LMO2, PPARD, RETNLB, and DSCAM, while the most activated were FN1, miR-125b-5p, ACOX1, RB1, and SIRT6 (Figure 6C). Fibronectin-1 (FN1) and fibronectin interacting proteins were altered by high pathogenic strains of Influenza virus [58]. The expression of miR-125b-5p was altered in HBV patients [59] and COVID-19 patients [60], and found important for replication of Japanese encephalitis virus [61] and Hepatitis C virus [62]. ACOX1 was downregulated by Enterovirus 71 infection in neural cells [63]. Retinoblastoma protein Rb (RB1) is a therapeutic potential target for oncolytic viruses [64,65]. SIRT6 acts as a negative regulator in dengue virus-induced inflammatory response [66], and a critical host factor for HBV replication [67]. However, the link between HSPA5 and the upstream regulation in IAV replication needs further investigation.

### 3.3. HSPA5 Protein Is Required for Maturation of IAV During Replication

In HSPA5 -deficient cells, NS1 protein levels were reduced during the early phase of IAV replication (Figure 4C). Although NP expression also appeared lower at early time points, this difference was not statistically significant. Interestingly, NP levels became significantly elevated at later stages of infection in HSPA5 knockdown cells (Figure 4D), which was further supported by immunofluorescence microscopy showing intracellular accumulation of NP proteins (Figure 4F). In contrast, viral RNA transcription remained unaffected by HSPA5 knockdown during IAV infection (Figure 4E). A previous study indicated that the IAV hemagglutinin protein is capable of direct interaction with HSPA5 and is involved in viral attachment and internalization [27]. Consistent with this finding, the current study also indicates that earlier steps in the IAV replication cycle, such as attachment and endocytosis, are affected by HSPA5 knockdown. The reduction of HSPA5 expression likely delayed or inhibited the attachment or internalization of the virus particle, consequently leading to the observed reduction in NS1 and NP expression during the early stages of infection (Figure 4C,D). Moreover, HSPA5 has similarly been found necessary for the attachment and entry of other viruses, including Porcine Epidemic Diarrhea Virus [68] and SARS-CoV-2 [69].

However, at the later stages of replication NP proteins were significantly higher in HSAP5 KD cells, but significantly lower number of progeny viruses in the supernatant was detected compared to the wild type cells. One of the main functions of HSPA5 is folding protein into appropriate structures. HSPA5 functions as a molecular chaperone within the endoplasmic reticulum (ER) lumen, where it regulates protein folding and ensures protein quality control. Its interaction with ERdj5, an ER-resident protein, facilitates both the proper folding of newly synthesized polypeptides and the degradation of misfolded proteins [70,71,72,73]. Beyond its chaperone role, HSPA5 acts as a key suppressor of the unfolded protein response (UPR) [74,75], contributes to the proteolytic processing of multiple proteins, and assists in the translocation of secretory proteins across the ER following translation [76].

**Figure 7:**
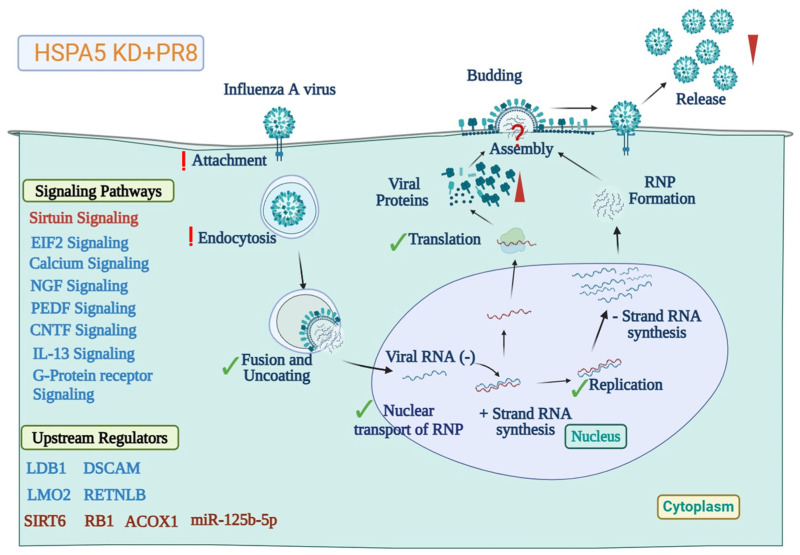
Schematic illustration of the proposed role of HSPA5 in the IAV replication cycle. The model summarizes the potential contribution of HSPA5 at different stages of the IAV life cycle. Steps unaffected by HSPA5 knockdown are indicated by green check marks, whereas processes likely disrupted by HSPA5 knockdown are marked with red question marks. The red exclamation sign shows the stages where HSPA5 is possibly involved but that need further investigation. The model of the IAV replication cycle was adapted from reference [79] and modified using BioRender (https://biorender.com).

Our data indicate that HSPA5 may be necessary for early stage/stages of virus replication but also for the maturation of the virions. In HSPA5-deficient cells, the folding of viral NP and NS1 proteins may be compromised, resulting in proteasomal degradation of misfolded NS1 and impaired incorporation of NP into maturated viral particles (Figure 7). Further studies are required to delineate the precise role of HSPA5 in facilitating viral protein folding and maturation.

The HSPA5 inhibitor Epigallocatechin-3-gallate (EGCG) also significantly reduced IAV replication in A549 (Figure 3C,D) and MRC5 cells (Figure 3E). However, EGCG exhibited differential cytotoxicity between A549 and MRC5 cells, which may depend on the antioxidant capacity of each cell type [77]. A549, a cancer cell line, has higher basal ROS levels than the normal lung fibroblast cell line MRC5. In contrast, an in vivo study in rats showed no evidence of genotoxicity at doses up to 2000 mg/kg or following intravenous administration of 50 mg/kg/day [78]. Thus, EGCG could serve as a potential antiviral agent against IAV infection but need further investigation to evaluate and eliminate the off-target effects.

## 4. Materials and Methods

### 4.1. Cells and Viruses

Human A549 lung epithelial cells were maintained in Dulbecco’s modified Eagle medium (DMEM; Gibco, Grand Island, NY, USA) supplemented with nonessential amino acids, sodium pyruvate, L-glutamine, and 10% fetal bovine serum (FBS; Thermo Fisher Scientific, Mississauga, ON, Canada), as previously described [80]. Human fetal lung fibroblast MRC-5 cells (ATCC, CCL-171) were cultured in Eagle’s minimum essential medium (EMEM; ATCC, catalog no. 30-2003) with 10% FBS. All cell cultures were kept at 37 °C in a humidified incubator with 5% CO_2_ and sub-cultured three times per week to sustain monolayers. IAV strains A/Mexico/INDRE4487/2009 (H1N1; pdm09), A/WSN/1933 (H1N1; WSN), and the mouse-adapted A/PR/8/34 (H1N1; PR8) were employed in the study. For virus amplification, MDCK cells were infected at a multiplicity of infection (MOI) of 0.01 plaque-forming units (PFUs) per cell, and culture supernatants were collected after 48 h. Viral stocks were concentrated by centrifugation at 64,000× *g* for 2 h at 4 °C, resuspended in phosphate-buffered saline (PBS) containing 10% glycerol, and stored in aliquots at −80 °C until further use.

### 4.2. Infection and Plaque Assay

Human A549 cells were cultured to approximately 70–80% confluence, rinsed twice with 1× PBS, and subsequently infected with IAV strains PR8, WSN, or pdm09. To evaluate the effect of HSPA5 knockdown on viral replication, infections were carried out at a multiplicity of infection (MOI) of 0.01. Culture supernatants were collected at multiple time points post-infection (0, 2, 4, 8, 16, 24, 36, and 45 hpi) for viral titration. Plaque assays were performed to quantify viral output. Briefly, supernatants were serially diluted tenfold in gel saline, and 100 μL of each dilution was inoculated in duplicate onto MDCK cell monolayers in 6-well plates. Plates were gently rocked for 1 h to facilitate adsorption, after which an overlay consisting of serum-free DMEM supplemented with 0.8% Avicel, 2 mM sodium pyruvate, 2 mM L-glutamine, 1× MEM nonessential amino acids, antibiotics (gentamicin and amphotericin B), and 2.5 μg/mL trypsin was added. Infected cells were incubated at 35 °C for 72 h. Following incubation, the overlay was removed, monolayers were washed with PBS and fixed with 2% formaldehyde for 30 min. Plaques were visualized by staining with crystal violet for at least 1 h, after which excess stain was removed, plates were dried, and plaques were enumerated. Viral titers were calculated as plaque-forming units (PFUs) per milliliter [80].

### 4.3. Cell Viability

Cell viability following HSPA5 knockdown was evaluated using the WST-1 assay (Roche, Mississauga, ON, Canada) according to the manufacturer’s instructions. A total of 8000 A549 cells were seeded per well in 96-well plates and allowed to adhere for 24 h at 37 °C. Cells were then transfected with either non-silencing scrambled (NSC) siRNA or HSPA5-specific siRNA. To assess viability at 48 and 72 h post-transfection, 9 μL of WST-1 reagent was added to each well, followed by incubation for 2 h at 37 °C. Absorbance changes were recorded using a microplate reader, and cell viability was expressed relative to NSC-transfected controls. Each experiment was repeated three times independently, with five technical replicates averaged for each biological replicate.

### 4.4. siRNA Transfection

Knockdown of HSPA5 protein expression was achieved using siRNA transfection as previously described [81]. Briefly, A549 cells were seeded in complete DMEM and transfected when they reached approximately 30–40% confluency. Prior to transfection, cells were washed twice with RNase-free PBS to remove FBS. Following the manufacturer’s instructions (Dharmacon, GE Healthcare, Lafayette, CO, USA), on-target (OT) and smart-pool (SP) siRNAs against HSPA5 (50 nM), along with NSC siRNA (50 nM), were prepared in Opti-MEM and complexed with DharmaFECT (Cat. #T-2001) for 20 min at room temperature. The siRNA DharmaFECT complexes were then added directly to the cells. Cultures were maintained at 37 °C with 5% CO_2_. To assess the effect of HSPA5 knockdown on IAV replication, cells were infected with IAV 48 h post-transfection (hpt). Cell lysates were collected at various time points up to 45 h post-infection (hpi) to analyze viral protein expression and RNA synthesis, while culture supernatants were harvested over the same period to evaluate progeny virus production.

### 4.5. Protein Extraction and Quantification

A549 cells grown in either 6-well plates or 60 mm culture dishes were transfected with siRNAs and subsequently infected with IAV at a multiplicity of infection (MOI) of 3 plaque-forming units (PFUs) per cell. At defined time points, both infected and mock-treated cells were collected by scraping, washed three times with ice-cold phosphate-buffered saline (PBS), and lysed in 60 µL of mammalian protein extraction reagent (M-PER; Cat. #78501, Thermo Fisher Scientific) supplemented with 1× HALT^®^ protease inhibitor cocktail (Cat. #78430, Thermo Fisher Scientific) using sonication. Lysates were clarified by centrifugation at 14,000× *g* for 10 min at 4 °C to remove insoluble material. Protein concentrations were measured with the BCA™ Protein Assay Kit (Pierce, Rockford, IL, USA) using bovine serum albumin (BSA) standards (Cat. #23208, Thermo Fisher Scientific).

### 4.6. SomaScan Analyses

To investigate how HSPA5 knockdown impacts IAV-induced proteomic changes, A549 cells were transfected with scrambled siRNA (NSC) or HSPA5 siRNA and infected with IAV-PR8 strain. Cell lysates from three biological replicate samples of four groups (NSC, NSC + PR8, HSPA5 KD, and HSPA5 KD + PR8) were collected at 24 hpi and analyzed using the SomaScan v1.3K platform, which simultaneously quantifies 1307 proteins. SOMAmer reagents were used to selectively capture target proteins, and signal intensities were measured as relative fluorescent units (RFUs) [29,82,83]. The RFU values for each protein correlated directly with their abundance in the original samples. Data were log_2_-transformed for downstream bioinformatic analysis, following the procedure as described previously [84].

### 4.7. Immunoblotting

Western blotting was carried out to assess viral and host protein expression, as described previously [80]. Protein samples (10–30 µg) obtained from experimental conditions were separated on 10% or 12% SDS-PAGE gels and transferred onto 0.2 µm nitrocellulose membranes. Membranes were probed with the following primary antibodies: anti-GAPDH (Cell Signaling, Danvers, MA, USA; Cat. 2118L), anti-β-Actin (Cell Signaling, Cat. 3700S), anti-HSPA5 (MilliporeSigma, Burlington, MA, USA; Cat. MABC675), and custom-generated mouse monoclonal antibodies against IAV NS1 and NP proteins [85]. Detection was performed using horseradish peroxidase (HRP)-conjugated secondary antibodies, either horse anti-mouse or anti-rabbit (Cell Signaling, Cat. 7076 and Cat. 7074, respectively). Protein signals were visualized with enhanced chemiluminescence (ECL) reagents and imaged on an Alpha Innotech FluorChemQ MultiImage III system. Band intensities were quantified with ImageJ software version 1.50i (NIH, Bethesda, MD, USA), and statistical analysis and data visualization were conducted using GraphPad Prism version 9.1.0.

### 4.8. RNA Extraction and Real-Time PCR

To assess the impact of HSPA5 knockdown on influenza vRNA transcription, A549 cells were infected with the PR8 strain of IAV at a multiplicity of infection (MOI) of 3 and harvested at 24 hpi. Following infection, cells were rinsed with ice-cold PBS, and total RNA was isolated using the RNeasy Mini Kit (QIAGEN, Venlo, Netherlands). Complementary DNA (cDNA) was synthesized from 250 ng of purified RNA using the GoScript™ Reverse Transcription System (Promega, Madison, WI, USA). Quantitative real-time PCR (qRT-PCR) was performed with the Platinum™ SYBR™ Green qPCR SuperMix-UDG kit (Thermo Fisher) in a 25 μL reaction volume consisting of 12.5 μL 2× SYBR™ Green SuperMix, 0.5 μL ROX Reference Dye, 0.5 μL of each primer (10 μM; sequences listed below), 6 μL nuclease-free water, and 5 μL of cDNA (10 ng). Each condition was analyzed using three independent biological replicates, with two technical duplicates per sample. Reactions were run on the QuantStudio™ 3 Real-Time PCR System (Applied Biosystems, Waltham, MA, USA) under the following cycling conditions: 50 °C for 2 min, 95 °C for 2 min, followed by 40 cycles of 95 °C for 15 s and 60 °C for 30 s. Cycle threshold (Ct) values were normalized to 18S rRNA expression and compared against non-silencing siRNA controls. Primer sequences were: PR8-HA (Fwd: CATTCCGTCCATTCAATCC; Rev: AACCATACCATCCATCTATC), PR8-NP (Fwd: AGAGGGTCGGTTGCTCACAA; Rev: TGGCTACGGCAGGTCCATA), and PR8-NS1 (Fwd: CTTCGCCGAGATCAGAAATC; Rev: TGGACCATTCCCTTGACATT).

### 4.9. Impact of HSPA5 Inhibitors on IAV Replication

The effect of Epigallocatechin-3-gallate (EGCG), a known HSPA5 inhibitor (Sigma-Aldrich, St. Louis, MO, USA; Cat# E4143), on IAV replication was investigated using A549 and MRC-5 cells. We followed the protocol as described before [86]. In summary, cells were exposed to a range of EGCG concentrations (0–1000 μM) for 48 h in serum-free medium to determine the maximum non-toxic dose, defined as maintaining greater than 80% cell viability. Cytotoxicity was assessed with the WST-1 assay. Following treatment, EGCG concentrations of 250 μM in A549 cells and 15.6 μM in MRC-5 cells preserved over 80% viability. For subsequent infection assays, two to three concentrations below the 20% cytotoxicity threshold were selected. Cells were pretreated with EGCG for 2 h, then infected with either PR8 or NCal IAV strains. After 1 h of viral adsorption, the inoculum was replaced with serum-free DMEM supplemented with the corresponding EGCG concentration, antibiotics (gentamicin, 100 μg/mL; amphotericin B, 100 μg/mL), and trypsin (2.5 μg/mL). Cultures were maintained at 37 °C, and supernatants were collected at 45 h post-infection (hpi) for viral titration by plaque assay.

### 4.10. Photomicrography

At 4 days post-transfection (dpt), A549 cells were imaged at 200× magnification using a Canon A700 digital camera (Canon, Ota City, Tokyo, Japan) to assess the effects of siRNA treatment. Captured images were imported into Microsoft PowerPoint (Microsoft, Redmond, WA, USA), where minor adjustments to brightness and contrast were applied, ensuring that the relative context of the images remained unaltered.

### 4.11. Immunofluorescent Microscopy

Approximately 4000 A549 cells were plated per well onto 6 mm Multi-Spot Slides (Fisher Scientific, Cat. No. 99-910-90, Waltham, MA, USA) and cultured for 24 h at 37 °C in DMEM supplemented with 10% FBS. Cells were then transfected with either 50 nM HSPA5-targeting siRNA or 50 nM non-NSC siRNA for 48 h, followed by infection with IAV strain PR8 at a multiplicity of infection (MOI) of 3. At 24 hpi, samples were washed five times with 1× PBS, fixed for 15 min in 4% paraformaldehyde, and subsequently washed again five times in PBS before permeabilization with 0.1% Triton X-100 for 5 min. Fixed and permeabilized cells were blocked overnight at 4 °C in 3% bovine serum albumin (BSA). Primary antibody incubation was carried out overnight at 4 °C using either anti-HSPA5 antibodies or an in-house produced IAV mouse anti-NS1 antibody [85]. After five PBS washes, cells were incubated in 0.2% Tween-20 (PBT) with Alexa Fluor 488-conjugated anti-rabbit secondary antibody for 1 h. Nuclei were counterstained with DAPI mounting medium, and fluorescence was visualized using a Zeiss Axio Observer Z1 inverted microscope (Oberkochen, Germany).

### 4.12. Statistical and Bioinformatics Analyses

Raw fluorescence (RFU) measurements for each protein obtained from SomaScan were first converted to log2 values. The delta log2 values were then calculated by subtracting the log2 expression levels of proteins in mock infected samples from those in PR8-infected samples. These delta log2 values were subsequently converted into fold-change values. Statistical significance of expression changes was assessed using two-tailed Student’s *t*-tests and Z-score analyses across three biological replicates. For downstream bioinformatic analyses in Ingenuity Pathway Analysis (IPA), proteins exhibiting significant expression alterations (*p* < 0.05 or Z-scores ≥ +1.96σ or ≤−1.96σ) with fold changes exceeding ± 1.3 were selected. Western blot band intensities were quantified using ImageJ software (version 1.51K; NIH, USA), and resulting numerical data were analyzed using one-way or two-way ANOVA in GraphPad Prism 6.0, with *p*-values < 0.05 considered statistically significant. We plotted the most impacted diseases & bio functions and canonical functions related pathways for the two conditions (NSC + PR8; HSPA5 KD + PR8) in dot plots using R package “ggplot2 (v 4.0.0)”. The top 20 upstream regulators were also plotted in a dot plot using the same tool. Overlap between conditions was visualized with a pairwise Venn diagram using R Package “VennDiagram (v 1.8.0)”, with the sets of significant functions (*p* < 0.05) and FC cut off < 1.3 per condition to report the unique and shared counts.

## 5. Conclusions

This study demonstrates that HSPA5 is an important host dependency factor for IAV replication. However, EGCG, a HSPA5 inhibitor significantly inhibits two different strains of Influenza in two different epithelial cell lines. One of the limitations of this study is that we used A549 cancer cell line, and mouse adapted PR8 strain. Although these models are widely employed because the cell line is permissive to IAV infection and the PR8 strain is safe to handle under BSL-2 conditions. Future studies across diverse cell lines, influenza subtypes, and in vivo models are necessary to confirm their therapeutic value.

Our findings also suggest that HSPA5 may support viral protein folding and assembly, and that inhibition of HSPA5 disrupts these processes. However, as HSPA5 interacts with numerous cellular proteins and signaling pathways, targeting it directly could carry risks of unwanted side effects. This highlights the importance of further work using HSPA5 knockout cell lines or animal models to evaluate its precise role in infection and therapeutic potential. Importantly, while HSPA5 has been implicated in the replication of several viruses, host proteins are multifunctional, and not all viruses rely on the same host factors. This complexity underscores the challenge of developing safe and effective antivirals that target host pathways. Thus, a deeper understanding of HSPA5’s role in IAV biology will be essential for designing interventions that maximize antiviral efficacy while minimizing adverse effects, ultimately advancing preparedness against future influenza outbreaks and pandemics.

## Figures and Tables

**Table 1:** Number of differentially regulated proteins in wild-type and HSPA5 KD cells upon PR8 infection.

Range of Fold Change	PR8	Total Significant	HSPA5 KD + PR8	Total Significant
	(Protein No.)	(Protein No.)	(Protein No.)	(Protein No.)
and F.C. > 1.00	76	218	277	503
and F.C. < 1.00	142		226	
and F.C. > 1.10	68	197	260	478
and F.C. < −1.10	129		218	
and F.C. > 1.20	43	144	170	347
and F.C. < −1.20	101		177	
and F.C. > 1.30	31	116	78	223
and F.C. < −1.30	85		145	
and F.C. > 1.50	20	68	23	112
and F.C. < −1.50	48		89	
and F.C. > 1.60	17	57	20	92
and F.C. < −1.60	40		72	
and F.C. > 2.00	11	33	12	40
and F.C. < −2.00	22		28	
and F.C. > 2.50	8	22	6	19
and F.C. < −2.50	14		13	

Significance was determined by T-test and Z-score as detailed in Materials and Methods from three biological replicates. The list of proteins dysregulated ≥ 1.5-fold in either direction is listed in Table 2.

**Table 2:** Proteins showing ≥ 1.5-fold changes in either direction are summarized.

Type(s)	Symbols	Entrez Gene Name	NSC + PR8 (FC)	*p*-Value	HSPA5 KD + PR8 (FC)	*p*-Value
Cytokine	CXCL8	C-X-C motif chemokine ligand 8	10.267	2.21 × 10^−5^	4.228	2.08 × 10^−4^
	CCL13	C-C motif chemokine ligand 13	−1.419	9.43 × 10^−2^	−2.282	2.29 × 10^−3^
	PF4	platelet factor 4	−1.261	1.02 × 10^−1^	−1.821	2.70 × 10^−3^
	IFNL1	interferon lambda 1	2.868	3.46 × 10^−2^	2.007	3.08 × 10^−3^
	CCL5	C-C motif chemokine ligand 5	6.386	3.59 × 10^−3^	5.011	3.87 × 10^−3^
	CCL20	C-C motif chemokine ligand 20	−1.275	2.61 × 10^−1^	−2.346	1.22 × 10^−2^
Enzyme	PARK7	Parkinsonism associated deglycase	1.102	4.97 × 10^−1^	1.993	1.34 × 10^−3^
	TOP1	DNA topoisomerase I	1.5	1.82 × 10^−3^	3.771	1.39 × 10^−3^
	HAT1	histone acetyltransferase 1	−2.37	2.10 × 10^−4^	−2.612	1.67 × 10^−3^
	CNTN1	contactin 1	−2.928	1.58 × 10^−2^	−2.908	1.70 × 10^−3^
	XRCC6	X-ray repair cross complementing 6	1.248	1.29 × 10^−1^	1.636	3.55 × 10^−3^
	EFEMP1	EGF containing fibulin extracellular matrix protein 1	−1.495	1.75 × 10^−2^	−1.619	8.03 × 10^−3^
	GNS	glucosamine (N-acetyl)-6-sulfatase	−1.223	7.69 × 10^−2^	−1.723	1.99 × 10^−2^
	ENTPD5	ectonucleoside triphosphate diphosphohydrolase 5	−1.49	4.88 × 10^−3^	−1.505	2.01 × 10^−2^
	AKR1A1	aldo-keto reductase family 1 member A1	−1.717	3.82 × 10^−2^	−1.619	2.03 × 10^−2^
	RNASEH1	ribonuclease H1	−1.129	5.80 × 10^−1^	−1.711	4.67 × 10^−2^
	PPIF	peptidylprolyl isomerase F	1.729	2.93 × 10^−2^	1.068	1.99 × 10^−1^
	PPID	peptidylprolyl isomerase D	−1.532	1.02 × 10^−2^	−1.532	2.33 × 10^−1^
Growth factor	PROK1	prokineticin 1	−1.288	1.64 × 10^−1^	−1.952	1.21 × 10^−3^
	FGF6	fibroblast growth factor 6	−1.63	5.64 × 10^−3^	−1.619	1.50 × 10^−3^
	DKK1	dickkopf WNT signaling pathway inhibitor 1	−3.605	5.46 × 10^−3^	−3.891	6.04 × 10^−3^
	BMP6	bone morphogenetic protein 6	−1.564	3.23 × 10^−2^	−1.575	7.89 × 10^−3^
	GRN	granulin precursor	−2.085	4.60 × 10^−3^	−1.765	1.05 × 10^−2^
	NRG1	neuregulin 1	−1.613	4.73 × 10^−2^	−1.49	2.68 × 10^−2^
	ANGPT2	angiopoietin 2	−1.235	2.46 × 10^−1^	−1.784	4.88 × 10^−2^
Kinase	PRKCG	protein kinase C gamma	−1.608	1.95 × 10^−2^	−1.664	6.01 × 10^−4^
	CKM	creatine kinase, M-type	−1.169	1.10 × 10^−1^	−1.521	8.87 × 10^−4^
	MET	MET proto-oncogene, receptor tyrosine kinase	−1.5	9.39 × 10^−2^	−2.848	9.85 × 10^−4^
	CDK2	cyclin dependent kinase 2	−1.257	2.15 × 10^−1^	−2.395	1.54 × 10^−3^
	EPHB2	EPH receptor B2	−1.079	2.10 × 10^−1^	−1.619	2.32 × 10^−3^
	FGFR1	fibroblast growth factor receptor 1	−2.071	3.29 × 10^−3^	−2.488	3.24 × 10^−3^
	EFNA2	ephrin A2	−1.659	4.84 × 10^−3^	−1.306	4.19 × 10^−3^
	INSR	insulin receptor	−1.429	2.23 × 10^−2^	−1.759	5.56 × 10^−3^
	ACVR1B	activin A receptor type 1B	−1.324	1.73 × 10^−1^	−2.056	7.77 × 10^−3^
	MAPK3	mitogen-activated protein kinase 3	−1.38	7.49 × 10^−2^	−1.619	8.63 × 10^−3^
	CHEK2	checkpoint kinase 2	−1.352	5.81 × 10^−2^	−1.873	1.27 × 10^−2^
	STC1	stanniocalcin 1	2.403	2.88 × 10^−3^	−1.619	1.40 × 10^−2^
	GRK2	G protein-coupled receptor kinase 2	−1.495	7.34 × 10^−2^	−1.505	1.41 × 10^−2^
	RPS6KA3	ribosomal protein S6 kinase A3	−1.532	6.31 × 10^−2^	−1.688	1.66 × 10^−2^
	MAPKAPK3	MAPK activated protein kinase 3	−1.67	6.14 × 10^−2^	−1.711	1.72 × 10^−2^
	AKT1	AKT serine/threonine kinase 1	−1.366	1.55 × 10^−1^	−1.63	1.81 × 10^−2^
	AKT3	AKT serine/threonine kinase 3	−1.366	1.55 × 10^−1^	−1.63	1.81 × 10^−2^
	CAMK2D	calcium/calmodulin dependent protein kinase II delta	−1.5	4.20 × 10^−2^	−1.597	2.54 × 10^−2^
	AKT2	AKT serine/threonine kinase 2	−1.495	5.46 × 10^−2^	−1.516	2.63 × 10^−2^
	EPHA2	EPH receptor A2	3	4.71 × 10^−3^	1.39	4.25 × 10^−2^
	PIK3CA	phosphatidylinositol-4,5-bisphosphate 3-kinase catalytic subunit alpha	−1.625	4.25 × 10^−2^	−1.803	4.38 × 10^−2^
	PIK3R1	phosphoinositide-3-kinase regulatory subunit 1	−1.625	4.25 × 10^−2^	−1.803	4.38 × 10^−2^
	ERBB3	erb-b2 receptor tyrosine kinase 3	−1.485	8.60 × 10^−2^	−1.873	4.39 × 10^−2^
	PDPK1	3-phosphoinositide dependent protein kinase 1	−1.537	6.06 × 10^−2^	−1.625	4.61 × 10^−2^
	CAMK2B	calcium/calmodulin dependent protein kinase II beta	−1.553	5.96 × 10^−3^	−1.537	4.87 × 10^−2^
	EPHA3	EPH receptor A3	1.602	2.03 × 10^−2^	1.046	2.68 × 10^−1^
Peptidase	PCSK9	proprotein convertase subtilisin/kexin type 9	−5.205	7.16 × 10^−3^	−6.084	1.24 × 10^−3^
	CMA1	chymase 1	−1.177	1.42 × 10^−1^	−1.532	1.91 × 10^−3^
	CTSA	cathepsin A	−2.107	4.83 × 10^−3^	−2.354	2.04 × 10^−3^
	CPE	carboxypeptidase E	1.169	3.73 × 10^−1^	1.516	1.59 × 10^−2^
	CASP3	caspase 3	−1.141	5.07 × 10^−1^	−1.63	2.33 × 10^−2^
Phosphatase	PTPN6	protein tyrosine phosphatase non-receptor type 6	−1.537	1.17 × 10^−3^	−1.495	1.48 × 10^−3^
	PON1	paraoxonase 1	−1.181	1.72 × 10^−1^	−1.664	1.19 × 10^−2^
Transcription regulator	TBP	TATA-box binding protein	−1.537	5.77 × 10^−2^	−1.853	1.57 × 10^−3^
	NACA	nascent polypeptide associated complex subunit alpha	2.107	1.80 × 10^−2^	1.625	1.73 × 10^−3^
	STAT6	signal transducer and activator of transcription 6	−1.664	2.05 × 10^−2^	−1.58	8.39 × 10^−3^
	HMGN1	high mobility group nucleosome binding domain 1	−1.068	6.46 × 10^−1^	1.892	9.84 × 10^−3^
	STAT3	signal transducer and activator of transcription 3	−2.092	7.86 × 10^−3^	−1.485	3.42 × 10^−2^
	SMAD2	SMAD family member 2	−1.619	3.48 × 10^−2^	−1.5	5.83 × 10^−2^
	HMGB1	high mobility group box 1	−1.505	1.38 × 10^−2^	−1.202	8.93 × 10^−2^
	AIP	aryl hydrocarbon receptor interacting protein	−1.553	1.13 × 10^−2^	−1.516	2.00 × 10^−1^
	EEF1B2	eukaryotic translation elongation factor 1 beta 2	1.141	4.88 × 10^−1^	2.189	1.28 × 10^−2^
	EIF4EBP2	eukaryotic translation initiation factor 4E binding protein 2	−1.439	3.52 × 10^−2^	−1.608	3.46 × 10^−2^
Transmembrane receptor	MICB	MHC class I polypeptide-related sequence B	−1.735	3.03 × 10^−2^	−2.428	2.75 × 10^−4^
	RTN4R	reticulon 4 receptor	−2.979	1.40 × 10^−2^	−2.77	4.62 × 10^−4^
	TNFRSF1A	TNF receptor superfamily member 1A	−3.918	5.43 × 10^−3^	−2.742	9.55 × 10^−4^
	PLXNB2	plexin B2	−1.329	3.82 × 10^−2^	−1.729	1.28 × 10^−3^
	GFRA1	GDNF family receptor alpha 1	−1.945	1.53 × 10^−2^	−2.014	2.20 × 10^−3^
	RELT	RELT TNF receptor	−1.49	2.12 × 10^−2^	−1.602	2.69 × 10^−3^
	TNFRSF10D	TNF receptor superfamily member 10d	3.63	1.02 × 10^−2^	3	2.86 × 10^−3^
	LIFR	LIF receptor subunit alpha	1.149	3.27 × 10^−1^	−1.676	2.92 × 10^−3^
	NRP1	neuropilin 1	−2.151	1.80 × 10^−2^	−2.969	3.49 × 10^−3^
	TNFRSF21	TNF receptor superfamily member 21	−2.523	8.55 × 10^−4^	−2.471	3.58 × 10^−3^
	KIR2DL4	killer cell immunoglobulin like receptor, two Ig domains and long cytoplasmic tail 4	−1.699	1.01 × 10^−2^	−1.809	4.25 × 10^−3^
	SFRP1	secreted frizzled related protein 1	−1.21	6.59 × 10^−2^	−2	5.01 × 10^−3^
	IL6ST	interleukin 6 cytokine family signal transducer	−1.206	5.44 × 10^−3^	−1.532	6.61 × 10^−3^
	B2M	beta-2-microglobulin	2.488	1.30 × 10^−4^	2.166	8.44 × 10^−3^
	PLAUR	plasminogen activator, urokinase receptor	1.866	8.83 × 10^−3^	1.454	9.80 × 10^−3^
	AGER	advanced glycosylation end-product specific receptor	−1.181	2.49 × 10^−1^	−1.602	1.82 × 10^−2^
	IGF1R	insulin like growth factor 1 receptor	−1.49	1.73 × 10^−1^	−1.809	2.30 × 10^−2^
	PGLYRP1	peptidoglycan recognition protein 1	−1.469	5.55 × 10^−2^	−1.575	2.39 × 10^−2^
	ITGB1	integrin subunit beta 1	1.125	6.18 × 10^−1^	1.636	3.43 × 10^−2^
transporter	ATP5PO	ATP synthase peripheral stalk subunit OSCP	1.591	2.67 × 10^−2^	2.403	3.19 × 10^−3^
	MCL1	MCL1 apoptosis regulator, BCL2 family member	−1.395	4.72 × 10^−2^	−1.608	3.45 × 10^−3^
	BPI	bactericidal permeability increasing protein	−1.608	4.50 × 10^−3^	−1.711	3.68 × 10^−3^
	ALB	albumin	−1.338	1.27 × 10^−1^	−1.886	3.71 × 10^−3^
	AFM	afamin	1.189	1.64 × 10^−1^	1.67	1.09 × 10^−2^
	SNX4	sorting nexin 4	−1.613	2.04 × 10^−2^	−1.879	1.06 × 10^−1^
Other	SPARC	secreted protein acidic and cysteine rich	1.202	5.39 × 10^−1^	1.558	2.44 × 10^−4^
	MICA	MHC class I polypeptide-related sequence A	−1.772	4.92 × 10^−2^	−2.196	4.85 × 10^−4^
	AMIGO2	adhesion molecule with Ig like domain 2	−2.621	1.93 × 10^−2^	−1.945	5.42 × 10^−4^
	GREM1	gremlin 1, DAN family BMP antagonist	−1.537	1.05 × 10^−2^	−1.5	1.24 × 10^−3^
	CCNA2	cyclin A2	−1.257	2.15 × 10^−1^	−2.395	1.54 × 10^−3^
	DYNLL1	dynein light chain LC8-type 1	−1.157	1.61 × 10^−1^	−1.58	1.95 × 10^−3^
	RSPO2	R-spondin 2	−1.653	1.75 × 10^−2^	−1.735	2.09 × 10^−3^
	ISG15	ISG15 ubiquitin like modifier	7.21	4.52 × 10^−3^	8.168	2.49 × 10^−3^
	FGF12	fibroblast growth factor 12	−1.197	1.23 × 10^−1^	−1.765	3.70 × 10^−3^
	CRISP3	cysteine rich secretory protein 3	−1.185	2.18 × 10^−1^	−1.84	3.72 × 10^−3^
	LAMA1	laminin subunit alpha 1	−4.377	1.17 × 10^−3^	−5.098	4.98 × 10^−3^
	LAMB1	laminin subunit beta 1	−4.377	1.17 × 10^−3^	−5.098	4.98 × 10^−3^
	LAMC1	laminin subunit gamma 1	−4.377	1.17 × 10^−3^	−5.098	4.98 × 10^−3^
	TIMP2	TIMP metallopeptidase inhibitor 2	−1.032	6.93 × 10^−1^	−1.664	5.19 × 10^−3^
	DKK4	dickkopf WNT signaling pathway inhibitor 4	−3.618	8.22 × 10^−3^	−3.482	5.93 × 10^−3^
	MFGE8	milk fat globule EGF and factor V/VIII domain containing	−2.014	4.16 × 10^−2^	−1.959	6.42 × 10^−3^
	KREMEN2	kringle containing transmembrane protein 2	1.165	3.25 × 10^−1^	1.659	6.87 × 10^−3^
	H2AZ1	H2A.Z variant histone 1	−2.078	1.21 × 10^−2^	−1.796	7.65 × 10^−3^
	SERPINE2	serpin family E member 2	−2.648	1.65 × 10^−4^	−2.258	7.96 × 10^−3^
	GSN	gelsolin	−1.613	1.79 × 10^−1^	−2.37	8.05 × 10^−3^
	CFH	complement factor H	−1.84	5.70 × 10^−3^	−1.564	8.60 × 10^−3^
	TNC	tenascin C	−1.161	1.23 × 10^−1^	−1.521	1.06 × 10^−2^
	UNC5D	unc-5 netrin receptor D	−4.317	9.53 × 10^−4^	−3.434	1.12 × 10^−2^
	RPS7	ribosomal protein S7	1.248	4.44 × 10^−1^	2.242	1.39 × 10^−2^
	APP	amyloid beta precursor protein	−1.879	1.78 × 10^−2^	−2.242	1.58 × 10^−2^
	LGALS8	galectin 8	−1.569	6.70 × 10^−2^	−1.532	1.78 × 10^−2^
	DSG1	desmoglein 1	1.094	5.43 × 10^−1^	1.532	1.86 × 10^−2^
	RPS3A	ribosomal protein S3A	1.31	3.53 × 10^−1^	2.403	2.16 × 10^−2^
	H1-2	H1.2 linker histone, cluster member	2.77	7.98 × 10^−3^	4.925	2.18 × 10^−2^
	SLITRK5	SLIT and NTRK like family member 5	−1.558	1.40 × 10^−2^	−1.591	2.67 × 10^−2^
	ITGA1	integrin subunit alpha 1	1.125	6.18 × 10^−1^	1.636	3.43 × 10^−2^
	SERPINE1	serpin family E member 1	5.979	3.82 × 10^−3^	1.366	3.47 × 10^−2^
	KIF23	kinesin family member 23	−2.63	8.74 × 10^−3^	−2.181	3.77 × 10^−2^
	MEPE	matrix extracellular phosphoglycoprotein	−1.454	4.26 × 10^−2^	−1.548	4.62 × 10^−2^
	IGFBP2	insulin like growth factor binding protein 2	1.809	2.12 × 10^−2^	1.206	5.13 × 10^−2^
	TGFBI	transforming growth factor beta induced	1.505	1.41 × 10^−2^	−1.113	6.83 × 10^−2^
	CD55	CD55 molecule (Cromer blood group)	1.591	5.56 × 10^−4^	1.206	7.88 × 10^−2^
	IGFBP6	insulin like growth factor binding protein 6	1.959	5.31 × 10^−4^	1.169	8.05 × 10^−2^
	CST3	cystatin C	1.972	3.32 × 10^−3^	−1.181	1.98 × 10^−1^

List of proteins with fold change up-regulated ≥ 1.5 or down-regulated ≤ −1.5 FC = fold change. In our previous study, we used the SOMAScan data for NSC + PR8 for a different analysis (Rashid et al., 2022 [7]). All proteins tested by SOMAScan with expression value below 1.5 or not significantly changed are listed in Table S1.

## Data Availability

The original contributions presented in this study are included in the article/supplementary material. Further inquiries can be directed to the corresponding author(s).

## Supplementary Materials

The following supporting information can be downloaded at: https://www.mdpi.com/article/10.3390/ijms262210998/s1.
